# Dynamic synergistic interplay between ovarian antioxidant defense and angiogenesis sustains high egg production in laying hens

**DOI:** 10.1186/s40104-026-01420-z

**Published:** 2026-05-03

**Authors:** Kailong Qin, Minglu Gao, Xiaoying Liu, Yanli Liu, Xiaojun Yang, Jiantao Yang

**Affiliations:** College of Animal Science and Technology, Northwest A&F University, Yangling, 712100 China

**Keywords:** Laying hens, Ovarian angiogenesis, Ovarian antioxidant

## Abstract

**Background:**

Follicular development in laying hens requires a balance between angiogenesis and redox status, yet their synergistic interplay across different production levels and physiological stages remains unclear. This study compared high-production (HP) and low-production (LP) hens at 50 and 75 weeks of age using morphological, antioxidant, angiogenic, and transcriptomic analyses. An acute tBHP-induced oxidative stress model was further employed to elucidate the temporal coupling between these systems.

**Results:**

HP hens exhibited significantly superior laying rate and FCR compared to LP hens at both 50 and 75 weeks. Morphologically, HP ovaries featured more hierarchical follicles, denser vascular networks, and reduced senescence (β-galactosidase) and apoptotic (TUNEL) signals. Mechanistically, HP ovaries showed significantly enhanced antioxidant capacity (increased T-AOC, GSH, and SOD; decreased MDA) and upregulated expression of antioxidant genes (e.g., *NOX1*, *SOD3*, *HSPB1*). Concurrently, HP ovaries displayed dense CD31-positive microvascular signaling, with significantly higher protein levels of VEGF and ANGPT1. Similarly, gene expression (e.g., *VEGFA*, *KDR*, *ANGPT1*, and *ITGA5*) was upregulated. Transcriptomic profiling revealed a functional transition: differentially expressed genes at 50 weeks were primarily enriched in immune and metabolic pathways, whereas at 75 weeks, enrichment shifted to extracellular matrix organization and angiogenesis. Co-enrichment analysis identified p53, FoxO, and VEGF as core regulatory pathways, highlighting *ITGA5* and *HSPB1* as key nodal genes. Finally, the tBHP challenge significantly increased ovarian ROS, triggering a synchronous, compensatory upregulation of antioxidant (NRF2, HIF1α) and angiogenic (VEGF, ANGPT1) factors.

**Conclusion:**

This study demonstrates that a synergistic antioxidant-angiogenesis axis is a critical mechanism supporting sustained high yield, offering theoretical insights for optimizing the ovarian microenvironment and extending the productive lifespan of laying hens.

**Graphical Abstract:**

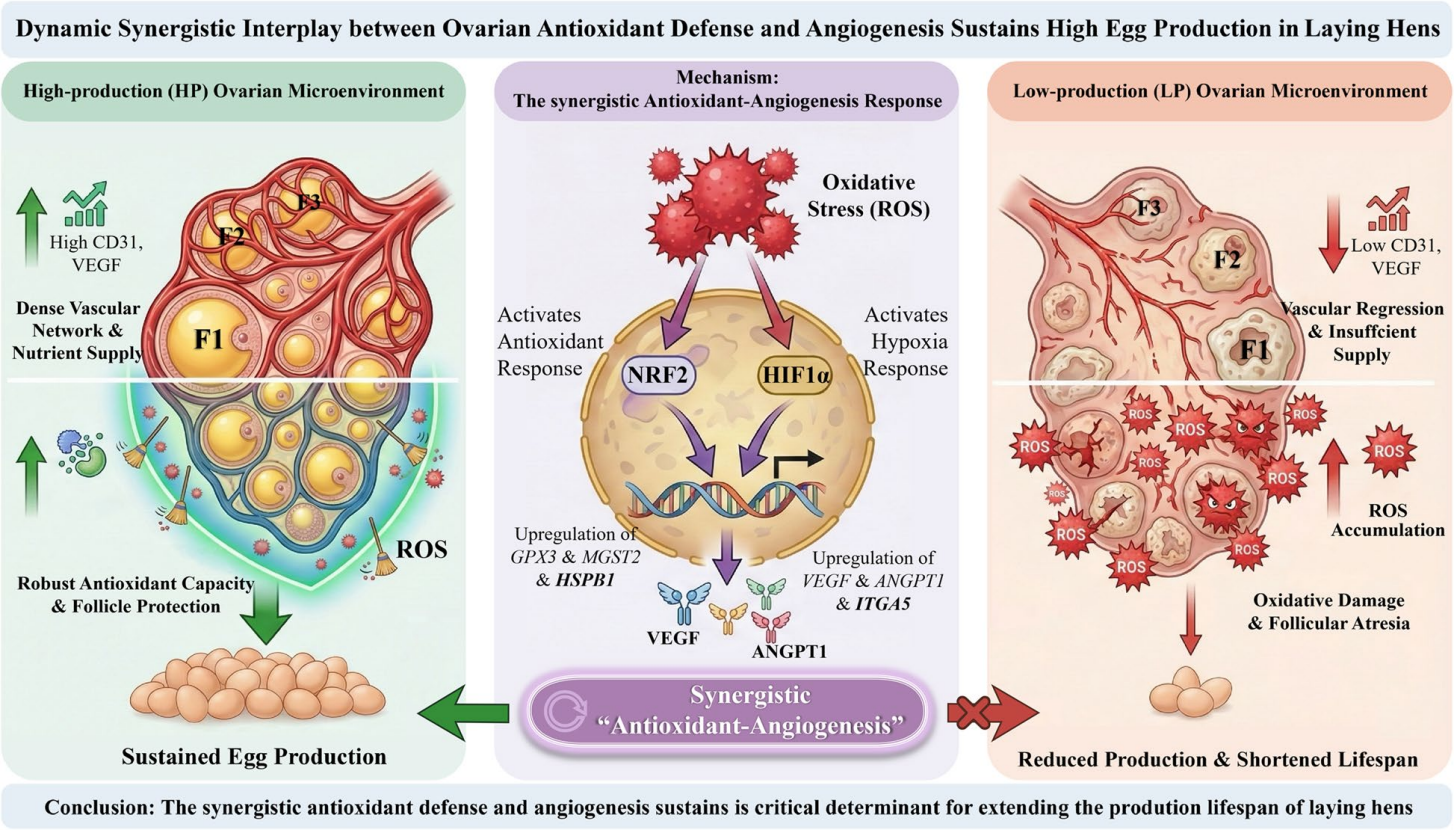

**Supplementary Information:**

The online version contains supplementary material available at 10.1186/s40104-026-01420-z.

## Background

The egg-laying performance of hens is a core determinant of economic benefits in poultry farming, and its sustainability is directly regulated by ovarian function [1, 2]. The ovary, as the physiological basis of follicular development, maintains efficient reproductive output through the precise coordination of endocrine signals, nutrient transport, and microenvironmental homeostasis [3–5]. However, even under identical husbandry conditions, marked variations in egg-laying performance exist among hens of the same age [6, 7]. The production cycle of modern commercial laying hens comprises distinct physiological phases. At 50 weeks of age, hens achieve physiological stability, combining full established body maturity with sustained peak egg production. Subsequently, 75 weeks of age represents a gradual physiological transition, serving as a key window to investigate the mechanisms that sustain reproductive persistence toward the industry’s “100-week, 500-egg” goal [8, 9]. In this context, elucidating the biological mechanisms underlying this disparity is crucial for understanding how to sustain high egg-laying performance.

Efficient follicular development in high-yield hens depends on adequate vascular supply and redox equilibrium [10–12]. On one hand, the ovary is highly vascularized [13], and the integrity of its vascular network is essential for follicular development [14, 15]. Angiogenesis plays a decisive role in follicular selection and maturation [16, 17]. On the other hand, the ovaries exhibit high metabolic activity and are susceptible to damage from reactive oxygen species (ROS) accumulation [18, 19]. Excessive oxidative stress accelerates follicular atresia, thereby impairing egg production [20–22]. Impaired angiogenesis or excessive oxidative stress can both lead to follicular atresia and ovarian dysfunction [19, 23, 24]. Thus, both adequate vascular supply and redox equilibrium are vital for maintaining ovarian function.

Notably, age-related functional decline in hens may involve a synergistic effect of reduced angiogenesis and diminished antioxidant capacity [10, 25]. Specifically, ovarian aging represents a progressive deterioration of the follicular microenvironment, characterized by the continuous accumulation of ROS and the concurrent regression of the microvascular network [26]. During this aging process, diminished antioxidant defenses fail to counter oxidative damage, while impaired angiogenesis restricts essential nutrient and oxygen delivery [27]. Therefore, this dynamic process provides a critical context for understanding why sustained reproductive performance depends on preserving both redox balance and adequate vascular supply. Growing evidence suggests a close association between angiogenesis and oxidative stress [28–31]. For example, hypoxia-inducible factor can simultaneously regulate vascular endothelial growth factor expression and redox balance [32, 33]. Conversely, continuous endothelial cell proliferation may itself lead to ROS accumulation [34, 35], accelerating ovarian functional decline. Although existing studies have separately elucidated the effects of oxidative stress and angiogenesis, their synergistic regulatory mechanism in high- and low-yield hens remains unclear, particularly the dynamic changes during the late laying period.

To address this knowledge gap, high- and low-production hens at peak (50 weeks) and late (75 weeks) laying stages were selected to conduct multi-level comparisons of ovarian antioxidant status and angiogenic characteristics. Furthermore, an oxidative stress model was established to dynamically observe its temporal effects on ovarian angiogenesis. This study aimed to: 1) analyze the respective advantages of ovarian antioxidant capacity and angiogenic potential in high-yield hens and explore the synergistic relationship between these two systems; 2) reveal the underlying biological basis that sustain superior laying performance by comparing ovarian function between high- and low- production hens at different laying stages; and 3) verify the temporal pattern of oxidative stress-induced compensatory angiogenesis. The findings will provide a theoretical basis for targeted improvement of the ovarian microenvironment and extension of the peak laying period.

## Methods

### Animals and experimental design

#### Ethics statement

The experimental procedures were approved by the Institutional Animal Care and Use Committee of Northwest A&F University (Permit No. NWAFAC 1008). All efforts were made to ensure the welfare of the animals and to provide a suitable environment.

#### Animals and management

**Fig. 1:**
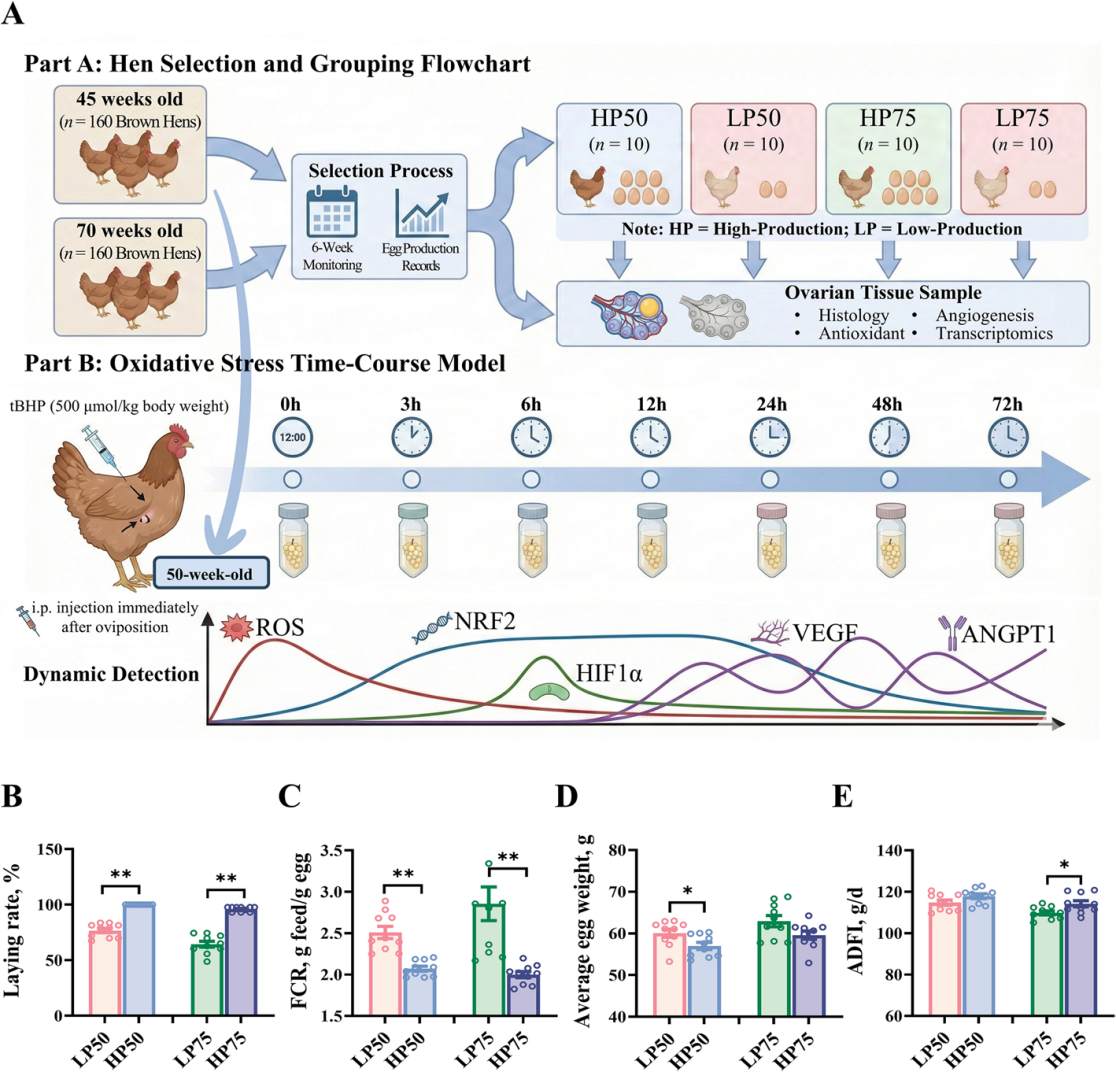
Experimental design and production performance of laying hens. **A** Experimental design. **B** Laying rate. **C** FCR. **D** Average egg weight. **E** ADFI. Data are expressed as mean ± standard error of the mean (SEM). * indicated a significant difference (*P* < 0.05), and ** indicated a highly significant difference (*P* < 0.01). FCR, a ratio of the feed intake to the egg weight; ADFI, average daily feed intake

This study was conducted at the Institute of Poultry-Crop Integration Systems, Northwest A&F University in Shaanxi Province, China. The experimental design is illustrated in Fig. 1A. A total of 360 healthy Hy-Line Brown hens were obtained from the same commercial farm. The flock consisted of 160 hens at 45-week-old (initial laying rate: 94.6% ± 5.8%; body weight: 1,920.6 ± 99.3 g) and 160 hens at 70-week-old (initial laying rate: 88.6% ± 8.7%; body weight: 2,008.2 ± 195.9 g). According to the Hy-Line Brown Management Manual and commercial flock performance data, the 45-week-old hens (post-body maturity) and 70-week-old hens (a physiological inflection point for the ‘100-week, 500-egg’ plan) were selected to represent stable peak production and late-laying functional decline, respectively [8, 36]. All hens were housed in a dedicated facility equipped with fully integrated smart environmental control systems, which maintained a constant ambient temperature of 22.0 ± 1.0 °C and relative humidity of 50.0% ± 5.0%, in line with standard commercial management practices. The lighting schedule consisted of 16 h of light and 8 h of darkness per day. Each hen was housed in an individual stainless-steel ladder cage (22 cm long, 40 cm wide, and 40–45 cm high) with ad libitum access to feed and water. A unified basal diet was provided to both age groups, with formulation and nutrient composition presented in Table S1.

#### Exp. 1: identification of high- and low-production hens and sample collection

To identify distinct production levels, individual egg numbers and egg weights were recorded for 6 consecutive weeks. Based on a normal distribution strategy, 10 high-production (HP) and 10 low-production (LP) hens were selected from each age group, forming the HP50, LP50, HP75, and LP75 groups. The HP groups comprised the top performers, representing optimal reproductive capacity. The LP groups consisted of individuals with laying rates 15%–25% below the group average. Extreme outliers were excluded to ensure the study focused on natural physiological divergence rather than pathological interference, as confirmed by the consistent performance trends shown in Fig. S1. Prior to sampling, hens were fasted overnight. Blood samples were collected from the wing vein, after which the hens were euthanized by exsanguination. Ovarian tissue was separated and collected; a portion was fixed in 4% paraformaldehyde solution or embedded in OCT compound, and the rest was stored promptly at −80 °C.

#### Exp. 2: tBHP-induced acute oxidative stress time-course model

To further investigate the effects of ovarian oxidative stress on the compensatory response to angiogenesis, 42 additional laying hens with similar body weight (1,989.5 ± 54.3 g) and laying rate (94.4% ± 2.6%) were selected from the 50-week-old cohort used in Exp. 1. These hens, considered to be at their peak physiological state, were selected as functionally sound baseline samples to characterize the maximum ovarian compensatory capacity under oxidative challenge. Immediately following oviposition (production of an egg), hens received an intraperitoneal injection of 4 mL tert-butyl hydroperoxide (tBHP, 500 μmol/kg body weight). Hens were euthanized at 7 time points post-injection: 0 h (pre-injection), 3 h, 6 h, 12 h, 24 h, 48 h, and 72 h (*n* = 6 per time point). Environmental parameters, dietary formulation, feeding management and sample collection procedures were strictly consistent with those described in Exp. 1.

### Ovarian morphology observation and tissue section staining

Ovarian tissues were collected and follicles were classified according to the follicle grade standards [1]. The ovarian tissue fixed for 24 h in 4% paraformaldehyde was embedded in paraffin and then sectioned into slices 4 μm thick. The hematoxylin and eosin (H&E) staining was performed using an H&E staining kit (C0105S, Beyotime, Shanghai, China) according to the manufacturer’s instructions, and images were captured and examined under a microscope. A TUNEL detection kit (Roche, Basel, Switzerland) was used to assess ovarian cell apoptosis. Ovarian paraffin sections were deparaffinized, washed twice in PBS, and then exposed to a TUNEL reaction mixture. After PBS rinses, the sections were counterstained with DAPI. The sections were observed and photographed using a fluorescence microscope. Blue fluorescence indicated background, while green fluorescence indicated apoptosis. Anti-CD31 antibody was used to evaluate angiogenic potential. The ovarian tissue sections were dewaxed, hydrated and antigen retrieved, then permeabilized and blocked with 5% BSA. The sections were treated with a diluted primary antibody against CD31 (A0378, ABclonal, Wuhan, China) and then incubated overnight at 4 °C. After washing with PBS, sections were exposed to secondary antibody for 1 h at room temperature. The sections were washed again with PBS, counterstained with DAPI, and mounted with an anti-fluorescence quenching mounting medium. Finally, the images were captured under a fluorescence microscope, CD31-positive markers (located on the vascular endothelial cell membrane) demonstrated green fluorescence (Alexa Fluor 488).

To assess β-galactosidase and reactive oxygen species (ROS) levels, ovarian tissues embedded in OCT compound were frozen-sectioned. SA-β-galactosidase activity was detected using the Senescence β-Galactosidase Staining Kit (C0602, Beyotime, Shanghai, China). The ovarian frozen section was rewarmed, washed in PBS, and then fixed in β-galactosidase staining fixative for 20 min at room temperature. After being thoroughly washed with PBS, the staining solution was added to cover the tissue, and the sections were incubated at 37 °C overnight in the dark. After incubation, observations were made under an ordinary light microscope. Some frozen sections were stained with dihydroethidium staining for ROS measurement. Specifically, sections were allowed to equilibrate for 30 min at room temperature. Then, the dihydroethidium fluorescent probe (S0064S, Beyotime, Shanghai, China), diluted in serum-free medium, was applied to cover the sections. After incubation for 30 min at 37 °C, sections were washed with pre-chilled PBS and mounted using anti‐fade mounting medium containing DAPI. ROS levels in the ovaries were observed under a fluorescence microscope (BX35, Olympus, Tokyo, Japan), and indicated by a red fluorescent signal.

### Antioxidant and angiogenesis markers

Serum was separated by centrifugation (3,000 × *g*, 10 min) in serum collection tubes. A 10% ovarian tissue homogenate was prepared by adding homogenate buffer with a weight to volume ratio of 1:9. The mixture was centrifuged for 10 min (4,000 × *g*) at 4 °C, and the supernatant was collected for later use. Antioxidant capacity analysis was performed using commercial kits (Nanjing Jiancheng Bioengineering Institute, Nanjing, China) according to the manufacturer's instructions. This included measuring the concentration of malondialdehyde (MDA) and total antioxidant capacity (T-AOC), as well as the activities of total superoxide dismutase (SOD), catalase (CAT), glutathione (GSH), and glutathione peroxidase (GSH-Px). Hydrogen peroxide (H_2_O_2_) and superoxide anion (O_2_⋅^−^) levels were assayed using Solarbio assay kits (Solarbio, Beijing, China). The content of nuclear factor E2-related factor 2 (NRF2; ED-69407), vascular endothelial growth factor (VEGF; ED-60148), angiopoietin 1 (ANGPT1; ED-63176), and hypoxia inducible factor 1 subunit alpha (HIF1α; ED-60538) in ovarian tissues was determined using ELISA kits ( LunChangShuoBiotech, Xiamen China).

### Ovarian gene expression by real-time PCR

Eight antioxidant-related genes (*NRF2*, *NOX1*, *HSPB1*, *SOD3*, *GPX3*, *MGST2*, *GSR*, and *HIF1α*) and six angiogenesis-related genes (*VEGFA*, *FLT1*, *KDR*, *ANGPT1*, *ITGA5*, *ECM1*) were analyzed using RT-qPCR. Gene-specific primers were designed based on *Gallus gallus* sequences (listed in Table S2). Total RNA of ovarian tissue was prepared using the TRIzol (AG21102, AG, Changsha, China). The concentration and quality of RNA were detected using the nucleic acid concentration analyzer NanoDrop 2000 (Thermo Fisher Scientific, Waltham, MA, USA). The cDNA was synthesized using the Primer Script RT Reagent Kit (TaKaRa, Dalian, China), following the manufacturer's recommended protocol. Then RT-qPCR was performed using SYBR green mix (Accurate Biology, Changsha, China) on the cDNA samples. The *β-actin* was used as an internal reference gene, and the relative expression of each target gene was calculated using the 2^−ΔΔCT^ method [37].

### Ovarian RNA-Seq experiment and data analysis

Transcriptome sequencing includes RNA extraction, detection, library construction, and sequencing. Ovarian tissue RNA was extracted using TRIzol (AG21102, AG, Changsha, China). Subsequently, total RNA was identified and quantified using a Qubit fluorescence quantifier (Life Technologies, CA, USA) and a Qsep400 high-throughput biofragment analyzer (Bioptic Inc., Taiwan, China). The qualified RNA samples were used to generate a library. After the initial library was constructed, the Qubit fluorescence quantifier and Qsep400 high-throughput biofragment analyzer were used to evaluate the library quality. The cDNA libraries were sequenced on the Illumina sequencing platform by Metware Biotechnology Co., Ltd. (Wuhan, China).

Raw data were filtered with FastQC (v0.23.2) [38], then the clean reads were aligned against the reference genome using HISAT2 (v2.2.1) [39]. Gene read counts were determined using featureCounts (v2.0.3) [40], and expression levels were normalized using the Fragments Per Kilobase of transcript per Million mapped reads (FPKM) method. DESeq (v1.22.1) [41, 42] was utilized for differential expression analysis, calculating fold change (FC) values between groups. Differentially expressed genes were identified based on the criteria |log₂FC|> 1 and *P*-value < 0.05. Principal component analysis (PCA) score plot, volcano map, Venn analysis, Kyoto Encyclopedia of Genes and Genomes (KEGG) analysis, and Gene Ontology (GO) enrichment analysis were performed using the Metware Cloud (https://cloud.metware.cn).

### Statistical analysis

Data analysis was performed using IBM SPSS 22.0 software. Independent samples *t*-tests were used to define significant differences between high and low laying hens. One-way ANOVA followed by Duncan's multiple comparison test was performed to evaluate the effects of ovarian oxidative stress on angiogenesis. All data were presented as arithmetic mean and SEM. Significance was declared at *P* < 0.05. Figures were created using GraphPad Prism 8.0 or Metware Cloud (https://cloud.metware.cn).

## Results

### Production performance

In the 50-week-old group (HP50 vs. LP50, Table S3), the HP50 group exhibited a significantly higher laying rate (Fig. 1B, *P* < 0.05) and a lower FCR (Fig. 1C, *P* < 0.05) compared to the LP50 group. Similar differences were also observed in the 75-week-old group (Table S4, *P* < 0.05). Conversely, average egg weight was significantly higher in the LP50 group than in the HP50 group (Fig. 1D, *P* < 0.05). For ADFI, values were significantly higher in the HP75 group compared to the LP75 group (Fig. 1E, *P* < 0.05), while no significant difference was observed between the LP50 and HP50 groups (*P* > 0.05). Furthermore, the consistent divergence in production performance observed over the 6-week screening period (Fig. S1) further validates the stability and reliability of the selected high- and low-production phenotypes.

### Ovarian morphological and histological characteristics

**Fig. 2:**
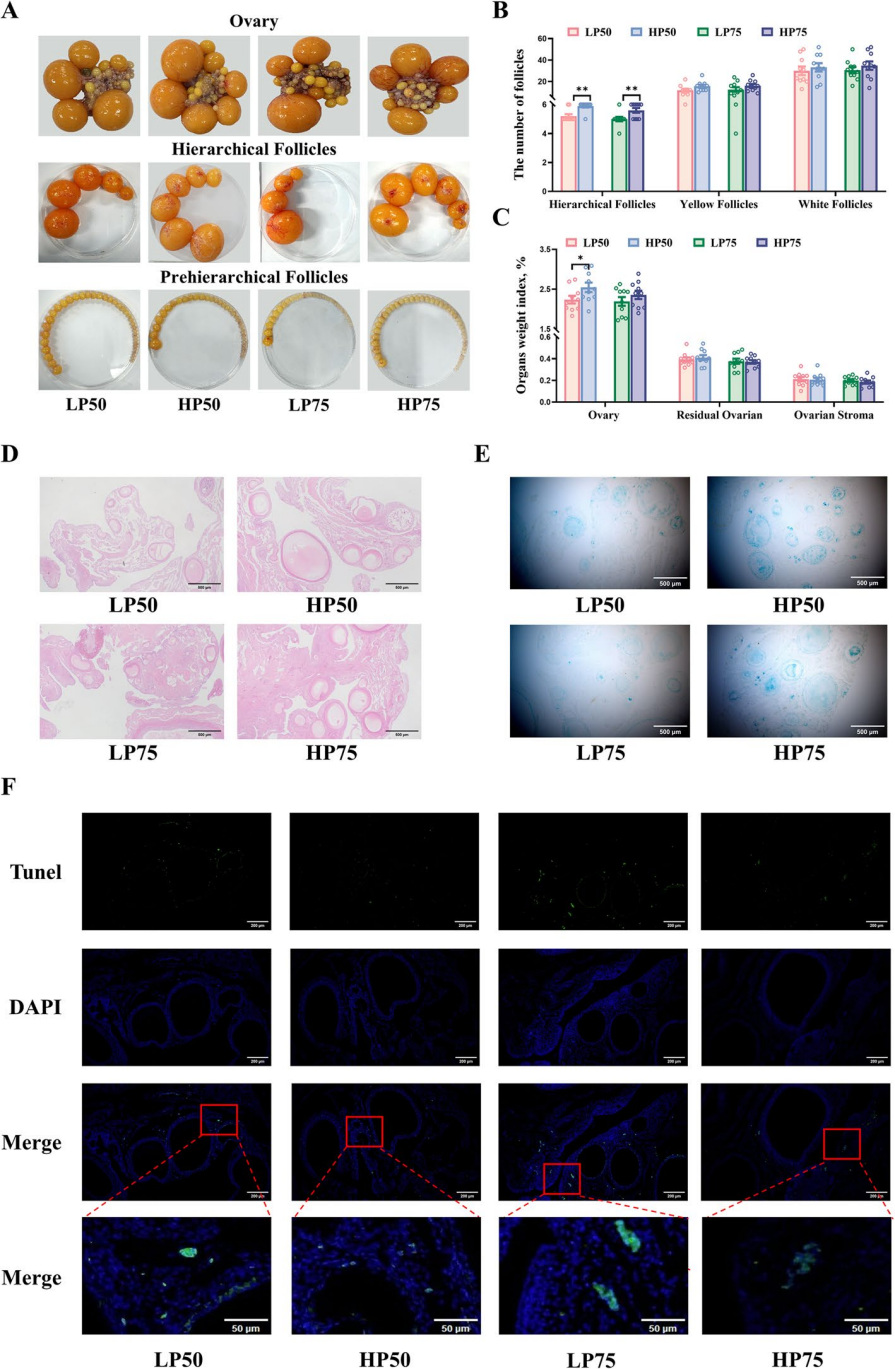
Ovarian morphological and histological characteristics of high- and low-yielding laying hens. **A** Photographs of the ovary and follicle. **B** The number of follicles. **C** Organ index. **D** Hematoxylin–eosin (H&E) staining of ovary. **E** β-galactosidase staining of ovary. **F** TUNEL staining of ovary. Data are expressed as mean ± standard error of the mean (SEM). * indicated a significant difference (*P* < 0.05), and ** indicated a highly significant difference (*P* < 0.01)

Macroscopically, the ovaries of HP50 and HP75 hens contained more developing follicles and exhibited a denser surface vascular network compared to their LP counterparts (Fig. 2A). Quantitative analysis confirmed a significantly higher count of hierarchical follicles in both HP groups (Fig. 2B, *P* < 0.05), whereas the numbers of yellow and white follicles, as well as the ovarian index showed no significant differences (Fig. 2B and C, *P* > 0.05). Histologically, H&E staining showed that numerous hierarchical follicles were present in the ovaries of the HP50 and HP75 groups (Fig. 2D). In contrast, primordial and atretic follicles were prominent in the LP groups. β-Galactosidase staining demonstrated widespread diffuse blue-positive signals in the theca layer, granulosa layer, and stroma of follicles from the LP groups (Fig. 2E). Concurrently, the TUNEL assay confirmed more intense TUNEL-positive staining in ovarian tissues from the LP groups compared to the HP groups (Fig. 2F).

### Antioxidant capacity

**Fig. 3:**
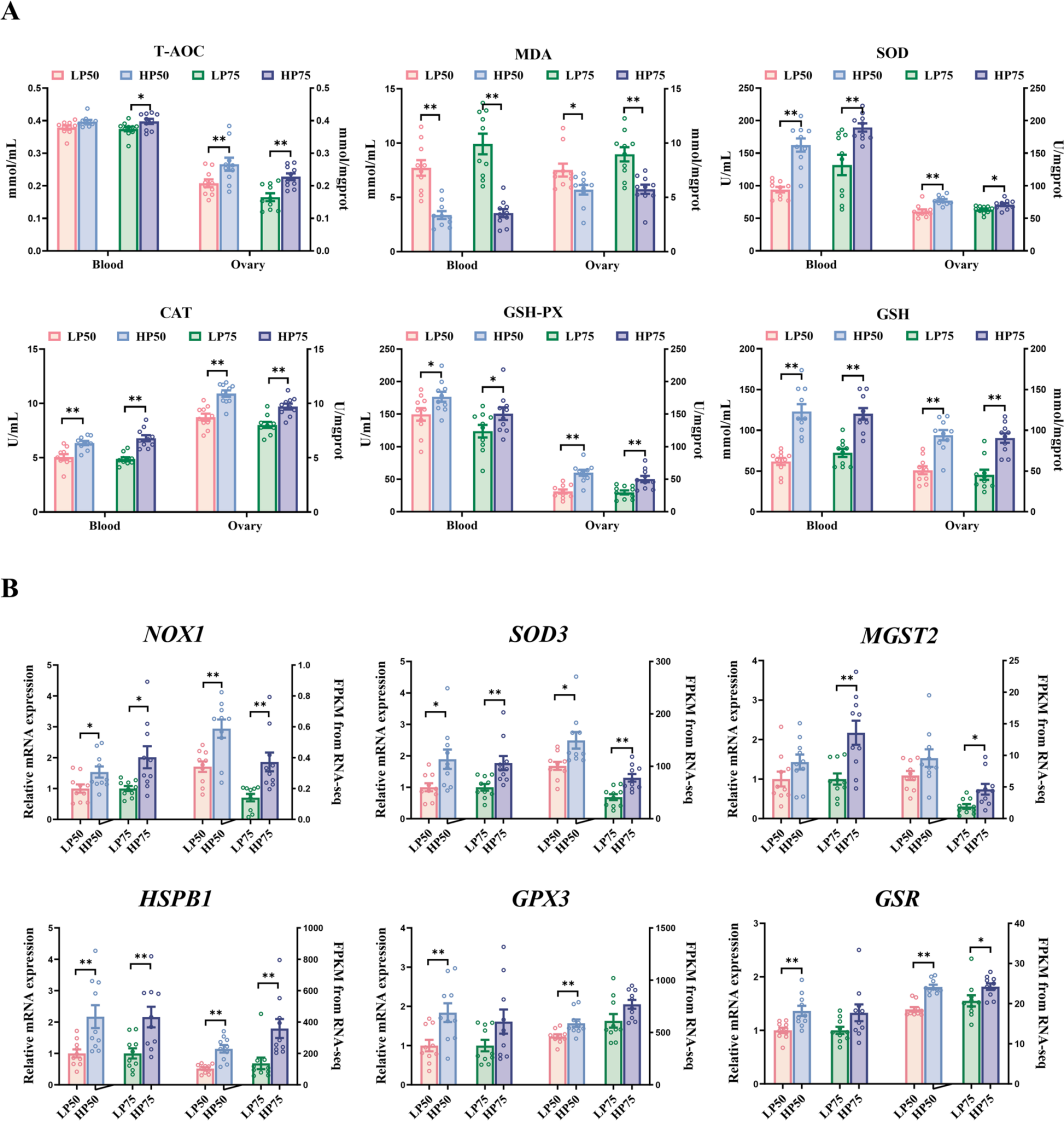
Antioxidant capacity of high- and low-yielding laying hens. **A** Antioxidant status of serum and ovary, including T-AOC, MDA, SOD, CAT, GSH-PX, GSH. **B** Antioxidant gene expression of ovary, including *NOX1*, *SOD3*, *MGST2*, *HSPB1*, *GPX3*, *GSR*. Data are expressed as mean ± standard error of the mean (SEM). * indicated a significant difference (*P* < 0.05), and ** indicated a highly significant difference (*P* < 0.01). T-AOC, total antioxidant capacity (expressed as Trolox equivalents); MDA, malondialdehyde; GSH, glutathione; GSH-PX, glutathione peroxidase; SOD, superoxide dismutase; CAT, catalase; *NOX1*, NADPH oxidase 1; *SOD3*, superoxide dismutase 3; *MGST2*, microsomal glutathione S-transferase 2; *HSPB1*, heat shock protein family B member 1; *GPX3*, glutathione peroxidase 3; *GSR*, glutathione-disulfide reductase

Figure 3 illustrates the differences in antioxidant capacity between the HP and LP groups. HP50 ovaries exhibited significantly higher levels of T-AOC and GSH (Fig. 3A, *P* < 0.05), along with significantly lower MDA content (*P* < 0.05), and the activities of SOD, CAT, and GSH-Px were significantly increased (*P* < 0.05). These antioxidant differences were consistently observed in the ovaries of the HP75 compared to the LP75 (*P* < 0.05). In blood parameters, the changes in antioxidant indicators (e.g., MDA, GSH, SOD, CAT, and GSH-Px) between HP and LP groups aligned with the patterns observed in ovarian tissues, with the exception of serum T-AOC in the 50-week-old group (Fig. 3A, *P* < 0.05). Regarding antioxidant-related genes, the mRNA expression of *NOX1*, *SOD3*, and *HSPB1* was significantly higher in HP50 and HP75 ovaries compared with their corresponding LP groups (Fig. 3B, *P* < 0.05), a result corroborated by transcriptomic sequencing data. Additionally, specifically upregulated genes included *GPX3* and *GSR* in the HP50 group, and *MGST2* in the HP75 group (*P* < 0.05).

### Angiogenesis capacity

**Fig. 4:**
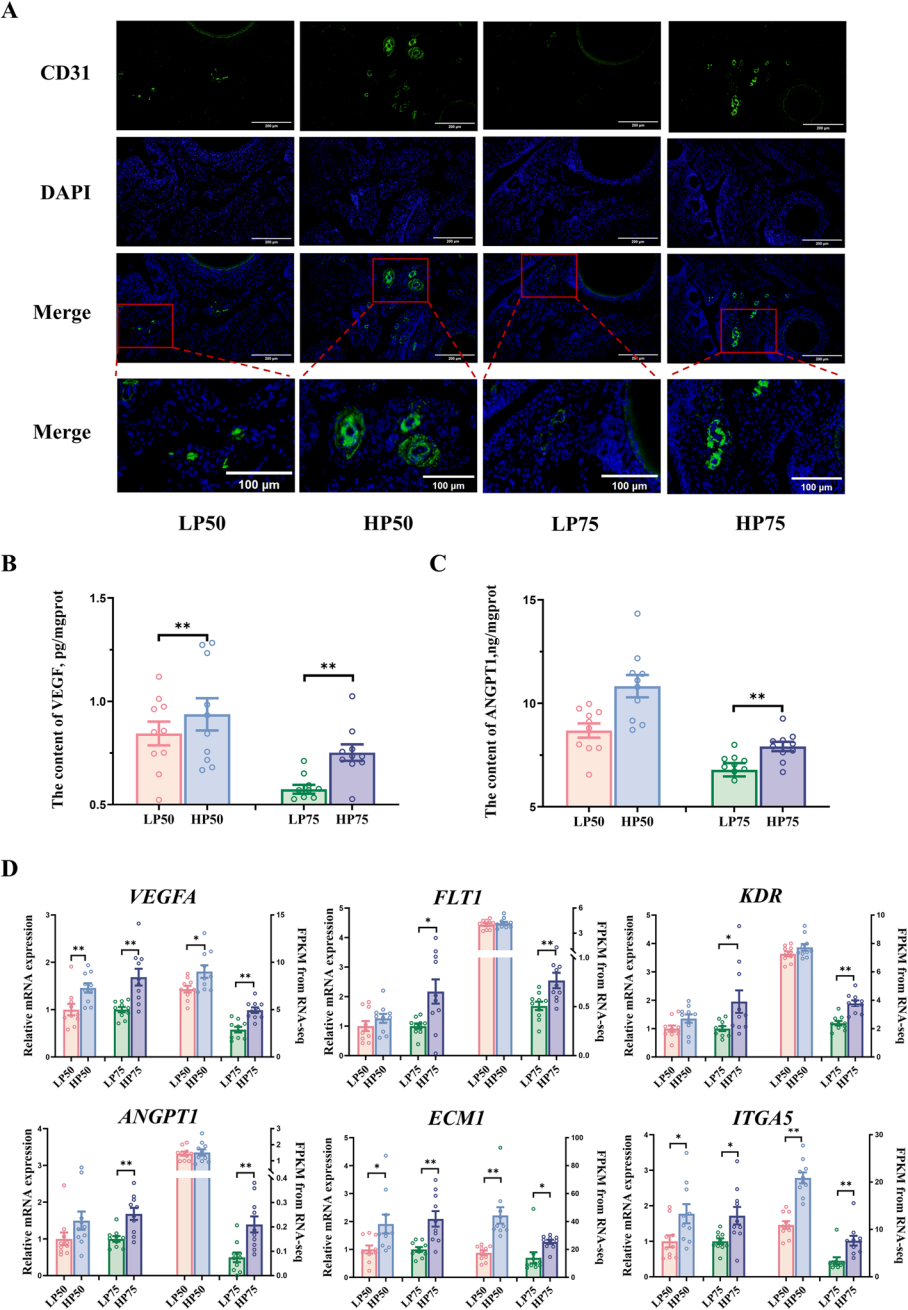
Angiogenesis capacity of high- and low-yielding laying hens. **A** CD31 staining of ovary. **B** The content of VEGF in ovary. **C** The content of ANGPT1 in ovary. **D** Angiogenesis gene expression of ovary, including *VEGFA*, *FLT1*, *KDR*, *ANGPT1*, *ECM1*, *ITGA5*. Data are expressed as mean ± standard error of the mean (SEM). * indicated a significant difference (*P* < 0.05), and ** indicated a highly significant difference (*P* < 0.01). CD31, platelet endothelial cell adhesion molecule-1; VEGF, vascular endothelial growth factor; ANGPT1, angiopoietin 1; *VEGFA*, vascular endothelial growth factor A; *FLT1*, vascular endothelial growth factor receptor 1; *KDR*, vascular endothelial growth factor receptor 2; *ANGPT1*, angiopoietin 1; *ECM1*, extracellular matrix protein 1; *ITGA5*, integrin alpha 5

Figure 4 shows the differences in angiogenesis capacity between the HP and LP groups. CD31 immunofluorescence staining revealed dense CD31-positive (green) signals at the ovarian cortex-medulla junction in the HP group, forming a rich tubular network (Fig. 4A). At the protein level, the VEGF content was significantly higher in HP50 ovaries than in LP50 (Fig. 4B, *P* < 0.05). Similarly, both VEGF and ANGPT1 content were significantly higher in HP75 ovaries than in LP75 (Fig. 4C, *P* < 0.05). At the mRNA level, multiple angiogenesis-related genes (*VEGFA*, *ECM1*, *ITGA5*) were significantly upregulated in the ovaries of both the HP50 and HP75 groups (Fig. 4D, *P* < 0.05), a finding supported by transcriptomic sequencing data. In addition, the expression levels of *FLT1*, *KDR*, and *ANGPT1* were also significantly higher in the HP75 group than in the LP75 group (*P* < 0.05).

### Ovarian transcriptome analysis

**Fig. 5:**
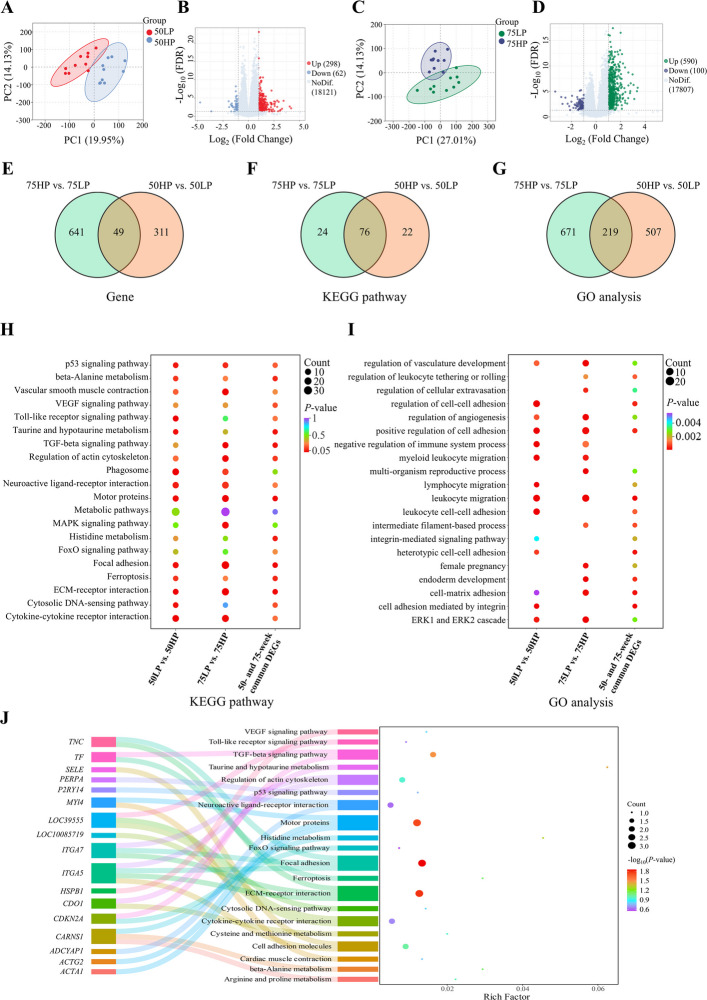
Ovarian transcriptome analysis of high- and low-yielding laying hens. **A** PCA score plot of 50-week-old laying hens. **B** Volcano map of 50-week-old laying hens. **C** PCA score plot of 75-week-old laying hens. **D** Volcano map of 75-week-old laying hens. **E** Venn analysis of DEGs. **F** Venn analysis of KEGG terms. **G** Venn analysis of GO terms. **H** Co-enrichment KEGG plot. **I** Co-enrichment GO plot. **J** Sankey bubble chart of DEGs in angiogenesis. PCA, principal component analysis; DEGs, differentially expressed genes; KEGG, Kyoto Encyclopedia of Genes and Genomes; GO, Gene Ontology

The ovarian transcriptomic sequencing results are shown in Fig. 5. PCA revealed clear separation between the HP50 and LP50 groups (Fig. 5A), as well as between the HP75 and LP75 groups (Fig. 5C), with samples clustering well within each group. Volcano plots identified 360 (298 up- and 62 down-regulated) and 690 (590 up- and 100 down-regulated) differentially expressed genes (DEGs) in the HP50 vs. LP50 (Fig. 5B) and HP75 vs. LP75 (Fig. 5D) comparisons, respectively. Enrichment analysis determined the significantly enriched pathways of the DEGs. KEGG enrichment analysis showed that in the 50-week-old group (Fig. S2A), DEGs were primarily enriched in immune response-related pathways (e.g., Toll-like receptor signaling pathway, NOD-like receptor signaling pathway) and metabolic processes (e.g., Drug metabolism-cytochrome P450, Cysteine and methionine metabolism). In the 75-week-old group (Fig. S2B), the significantly enriched pathways were mostly related to cell structure and tissue function, including Focal adhesions, ECM-receptor interaction, Vascular smooth muscle contraction, and Regulation of actin cytoskeleton, etc. This trend was further supported by GO enrichment analysis (Fig. S3). For the 50-week-old group, the DEGs were mainly involved in defense responses to bacterium, humoral/adaptive immune responses, and collagen binding, etc. For the 75-week-old group, they were significantly enriched in terms related to tissue structure, such as extracellular matrix organization, collagen fibril organization, blood circulation, and angiogenesis.

Venn analysis identified 49 common DEGs (Fig. 5E), 76 KEGG pathways (Fig. 5F), and 219 GO terms (Fig. 5G) between the HP50 vs. LP50 and HP75 vs. LP75. The co-enrichment analysis results showed significant enrichment in KEGG pathways related to angiogenesis and cell structure (Fig. 5H, e.g., VEGF signaling pathway, Vascular smooth muscle contraction, Focal adhesion, ECM-receptor interaction), as well as stress response and immune regulation (Fig. 5H; e.g., p53 signaling pathway, Toll-like receptor signaling pathway, FoxO signaling pathway, MAPK signaling pathway). Complementary GO analysis revealed significant enrichment including leukocyte/lymphocyte migration, regulation of vasculature development, regulation of angiogenesis, cell–matrix adhesion, and cell adhesion mediated by integrin (Fig. 5I). A Sankey-bubble plot showed the core DEGs and their regulatory relationships within key KEGG pathways (Fig. 5J). *CDKN2a* was involved in regulating the p53 signaling pathway, *ITGA5* and *ITGA7* mediated ECM-receptor interactions and Focal adhesion, and *HSPB1* regulated angiogenesis by participating in the VEGF signaling pathway.

### Temporal changes in angiogenesis under oxidative stress

**Fig. 6:**
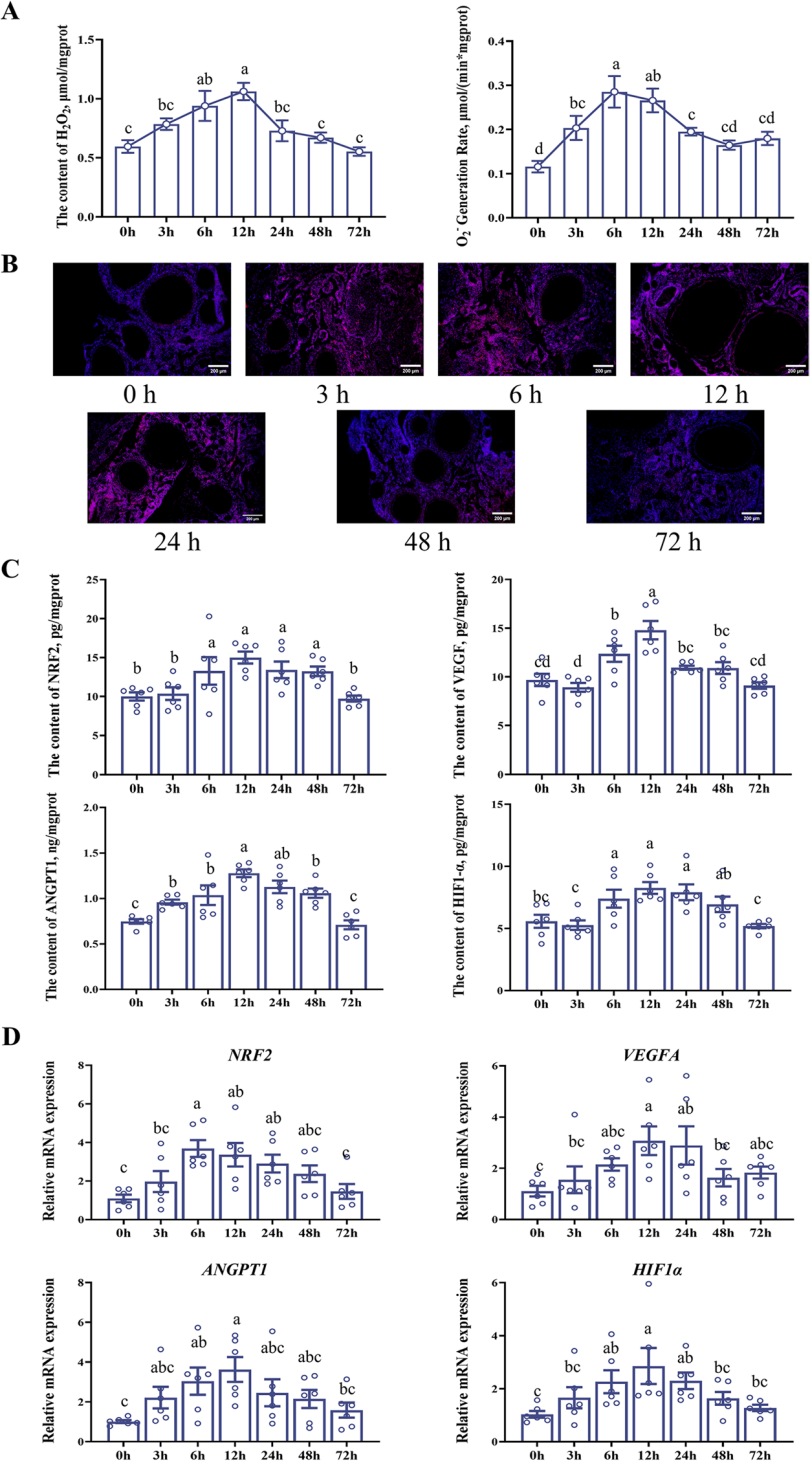
Temporal changes in ovarian angiogenesis under oxidative stress. A total of 42 high-yielding hens from Exp.1 (50-week-old, laying rate: 94.4% ± 2.6%) was injected with tBHP, and ovarian tissues were sampled at 7 time points (*n* = 6 per time point). **A** ROS levels of ovary, including the content of H_2_O_2_ and O_2_^−^ generation rate. **B** ROS staining of ovary, merged with DAPI-stained nuclei. **C** Antioxidant and angiogenesis status of ovary, including NRF2, VEGF, ANGPT1, and HIF1α. **D** Antioxidant and angiogenesis gene expression of ovary, including *NRF2*, *VEGFA*, *ANGPT1*, and *HIF1α*. Data are expressed as mean ± standard error of the mean (SEM). ^a–d^Different superscripts in a row indicate a significant difference (*P* < 0.05). ROS, reactive oxygen species; NRF2, nuclear factor E2-related factor 2; VEGF, vascular endothelial growth factor; ANGPT1, angiopoietin 1; HIF1α, hypoxia inducible factor 1 subunit alpha

Figure 6 depicts temporal changes in ROS levels and the expression of antioxidant- and angiogenesis-related proteins and genes in ovarian tissues of 50-week-old hens (from Exp. 1) at different time points after tBHP injection. The detection of ROS levels revealed significant differences in both H_2_O_2_ content and O_2_·^−^ generation rate at each time point (Fig. 6A, *P* < 0.05). Specifically, H_2_O_2_ content increased gradually post-injection, peaked at 12 h, and subsequently declined. The O_2_·^−^ generation rate peaked at 6 h and then decreased. Correspondingly, ROS fluorescence staining showed that the signal intensity increased significantly from 3 to 12 h post-injection and gradually decreased from 48 to 72 h (Fig. 6B). At the protein level, the contents of NRF2, VEGF, ANGPT1, and HIF1α all changed significantly over time (Fig. 6C, *P* < 0.05). Both antioxidant-related proteins (NRF2, HIF1α) and angiogenesis-related proteins (VEGF, ANGPT1) exhibited a similar dynamic pattern of initial induction followed by a decline. The mRNA expression of *NRF2*, *VEGFA*, *ANGPT1* and *HIF1α* at each time point was generally consistent with the trend of protein level changes (Fig. 6D, *P* < 0.05).

## Discussion

Through multi-level comparisons of ovarian function between high- and low-yield hens at peak and late laying stages, this study identifies key characteristics underpinning superior reproductive performance. Core findings demonstrate a coupled enhancement of antioxidant defenses and angiogenic potential in the ovarian microenvironment of high-yield hens. Moreover, an acute oxidative stress model confirmed that the ovary mounts a synchronous upregulation of both antioxidant and angiogenic factors in response to oxidative challenge, suggesting an interlinked regulatory mechanism between redox balance and vascular remodeling in maintaining ovarian function. In summary, these findings demonstrate that high-yield hens support sustained high egg production through a coordinated enhancement of ovarian antioxidant defense and angiogenic capacity.

Antioxidant capacity emerged as a key characteristic distinguishing high-yield from low-yield hens. Oxidative stress is a major contributor to diminished ovarian reserve (DOR) and premature ovarian insufficiency (POI) [43, 44]. Excessive ROS attack granulosa cells, thereby inducing mitochondrial dysfunction, accelerating apoptosis, and impairing hormone synthesis, which ultimately leads to ovarian function decline [45, 46]. Ovarian tissues of high-yield hens exhibited higher levels of SOD, CAT, GSH-Px, and GSH, alongside lower MDA content, compared to low-yield hens. This advantage was corroborated at the transcriptional level by the significantly upregulated expression of genes including *NOX1*, *GPX3*, and *HSPB1*. Excessive oxidative stress accelerates follicular atresia and compromises ovarian function [47, 48]. The widespread presence of senescence-associated β-galactosidase activity [49] and TUNEL-positive apoptotic cells [50] in the ovaries of low-yield hens, coupled with morphological observations showing increased numbers of primordial and atretic follicles, confirmed that accumulated oxidative stress causes tissue damage and functional decline, thereby significantly reducing laying performance [43, 45, 47].

Crucially, this antioxidant advantage does not act in isolation. Given that oxidative stress impairs vascular function [51, 52], our study confirms that the superior antioxidant capacity in high-laying hens is closely linked to a more developed ovarian vascular network. Ovarian tissues from high-laying hens exhibited a significantly denser vascular network and higher expression of angiogenesis-related factors (e.g., VEGFA, ANGPT1) at both protein and gene levels. A genome-wide studies have already identified angiopoietin-like protein 2 (*ANGPTL2*) as a key genetic regulator of egg-laying performance in chickens [53]. Collectively, these findings indicate that high-laying hen ovaries possess greater angiogenic potential. Interestingly, nutritional interventions employing plant extracts (e.g., *Angelica sinensis, Ligusticum chuanxiong*) with known antioxidant properties can activate angiogenesis-related pathways in laying hens [11, 54]. This supports our central hypothesis: efficient ovarian antioxidant defense mitigates oxidative damage, while active angiogenesis ensures adequate nutrient and oxygen supply; together, these processes synergistically sustain high production in high-yielding hens.

Furthermore, this synergistic interaction between antioxidant activity and angiogenesis exhibited dynamic changes with increasing age. The mechanisms underlying age-related ovarian decline were stage-specific [51, 55, 56]. At 50 weeks, differentially expressed genes were primarily enriched in immune response and metabolic pathways, suggesting that high-yielding hens sustain ovarian health via enhanced immune surveillance and metabolic efficiency. By 75 weeks (late laying stage), differentially expressed genes were significantly enriched in pathways involving extracellular matrix-receptor interactions, angiogenesis, and cytoskeleton regulation. This shift from metabolic and immune regulation to tissue structure maintenance particularly the vascular network and stromal support, highlights the growing importance of maintaining ovarian structural integrity for sustaining high yield as hens age [57–59]. Co-enrichment analysis further identified core pathways involved in oxidative stress responses (e.g., p53, FoxO, and MAPK signaling pathways) and angiogenesis regulation (notably the VEGF signaling pathway). Among these, key genes like *ITGA5* [60] and *HSPB1* [61] may act as critical nodes regulating sustained egg production.

To directly probe the dynamic relationship between the antioxidant and angiogenic systems, we established an oxidative stress model by injecting tBHP. This approach provided the first temporal evidence of how oxidative stress influences ovarian angiogenesis. Following tBHP-induced rapid ROS accumulation, the expression of stress-responsive transcription factors (NRF2, HIF1α) and angiogenic factors (VEGF, ANGPT1) displayed a dynamic pattern: an initial increase followed by a decline. NRF2 is a master regulator of the antioxidant response [62], whereas VEGF and ANGPT1 promote tissue repair and functional maintenance by enhancing local perfusion [63, 64]. Notably, HIF1α induction coincided temporally with the ROS peak. Given that HIF1α concurrently regulates VEGF expression and redox balance [65–67], this finding strongly suggests that the ovarian antioxidant and angiogenic systems are interconnected, forming a synergistic functional module. This insight offers a novel perspective on ovarian functional decline: persistent oxidative stress may ultimately exhaust or impair this compensatory capacity, leading to inadequate angiogenesis and follicular developmental disorders.

The current study presents several limitations. First, the physiological effects and intensity of chronic stresses experienced by laying hens throughout the production cycle require further exploration. Second, the specific molecular links connecting antioxidant defense and angiogenesis require validation through functional assays, such as gene knockdown or overexpression. Finally, the causal relationships between the identified core genes (e.g., *HSPB1*, *ITGA5*) and their regulatory networks require confirmation through subsequent experiments.

In summary, this study presents the first systematic investigation into the mechanisms supporting ovarian function from the perspective of antioxidant defense and angiogenesis synergistic regulation in high-yielding laying hens. The findings offer novel insights for extending the laying cycle by targeting the ovarian microenvironment, specifically through enhancing antioxidant defenses and promoting angiogenesis. Future research should elucidate the interplay among key nodal factors such as HSPB1, ITGA5, and the NRF2-HIF1α-VEGF axis, and explore their potential in mitigating ovarian decline.

## Conclusion

This study provides the first systematic evidence that the superior laying performance in high-yield hens is mediated by a synergistic improvement in ovarian antioxidant capacity and angiogenic potential, with this advantage becoming increasingly pronounced with advancing age. Moreover, the observation that oxidative stress induces a compensatory angiogenesis underscores the critical dynamic interplay between redox balance and vascular remodeling in maintaining ovarian function. Collectively, our results posit that targeting the antioxidant-angiogenesis axis is a viable strategy to optimize the ovarian microenvironment and prolong the laying period in aging hens.

## Supplementary Information

Additional file 1: Table S1. Ingredients and nutrient levels of the basal diet. Table S2. Primers used for RT-qPCR. Table S3. Comparison of production performance between high- and low-producing laying hens at 50 weeks of age. Table S4. Comparison of production performance between high- and low-producing laying hens at 75 weeks of age. Fig. S1. Dynamic production performance of high- and low-production laying hens during the 6-week screening period. Fig. S2. KEGG pathway enrichment analysis of DEGs. The group of HP50 vs. LP50. The group of HP75 vs. LP75. Fig. S3. GO enrichment analysis of DEGs. The group of HP50 vs. LP50. The group of HP75 vs. LP75.

## Data Availability

The composition and nutritional profiles of the experimental diets, along with the forward and reverse primer sequences used for PCR analysis, are provided in the Supplementary Materials. Further details regarding the transcriptomic analysis are also included therein.

## Abbreviations

ADFI: Average daily feed intake
ANGPT1: Angiopoietin 1
CAT: Catalase
CD31: Platelet endothelial cell adhesion molecule-1
DEGs: Differentially expressed genes
ECM1: Extracellular matrix protein 1
FC: Fold change
FCR: A ratio of the feed intake to the egg weight
FLT1: Vascular endothelial growth factor receptor 1
FPKM: Fragments per kilobase of transcript per million mapped reads
GO: Gene Ontology
GPX3: Glutathione peroxidase 3
GSH: Glutathione
GSH-PX: Glutathione peroxidase
GSR: Glutathione-disulfide reductase
H&E: Hematoxylin and eosin staining
H_2_O_2_: Hydrogen peroxide
HIF1α: Hypoxia inducible factor 1 subunit alpha
HP: High production
HSPB1: Heat shock protein family B member 1
ITGA5: Integrin alpha 5
KDR: Vascular endothelial growth factor receptor 2
KEGG: Kyoto Encyclopedia of Genes and Genomes
LP: Low production
MDA: Malondialdehyde
MGST2: Microsomal glutathione S-transferase 2
NOX1: NADPH oxidase 1
NRF2: Nuclear factor E2-related factor 2
O_2_⋅^−^: Superoxide anion activity
OCT: Optimal cutting temperature compound
PCA: Principal Component Analysis
ROS: Reactive oxygen species
SOD: Superoxide dismutase
SOD3: Superoxide dismutase 3
T-AOC: Total antioxidant capacity
tBHP: Tert-butyl hydroperoxide
VEGF: Vascular endothelial growth factor
VEGFA: Vascular endothelial growth factor A
