# Supplementary material for: Dynamic synergistic interplay between ovarian antioxidant defense and angiogenesis sustains high egg production in laying hens
Source: J Anim Sci Biotechnol. 2026 May 3;17:81. doi: 10.1186/s40104-026-01420-z (PMC13137499; doi:10.1186/s40104-026-01420-z)
Supplement: Supplementary file 1 — Additional file 1: Table S1. Ingredients and nutrient levels of the basal diet. Table S2. Primers used for RT-qPCR. Table S3. Comparison of production performance between high- and low-producing laying hens at 50 weeks of age. Table S4. Comparison of production performance between high- and low-producing laying hens at 75 weeks of age. Fig. S1. Dynamic production performance of high- and low-production laying hens during the 6-week screening period. Fig. S2. KEGG pathway enrichment analysis of DEGs. The group of HP50 vs. LP50. The group of HP75 vs. LP75. Fig. S3. GO enrichment analysis of DEGs. The group of HP50 vs. LP50. The group of HP75 vs. LP75. [file 40104_2026_1420_MOESM1_ESM.docx]

**Table S1** Ingredients and nutrient levels of the basal diet (as feed basis)

| **Item** | **% (unless noted)** |
| --- | --- |
| Ingredients |  |
| Corn | 59.76 |
| Soybean meal | 23.00 |
| Distillers dried grains with solubles | 4.00 |
| Calcium carbonate | 8.65 |
| Dicalcium phosphate | 1.05 |
| Soybean oil | 1.25 |
| Sodium chloride | 0.25 |
| L-Lysine-H2SO4 | 0.13 |
| L-Threonine | 0.31 |
| DL-Methionine | 0.25 |
| Choline chloride | 0.10 |
| Sand | 0.25 |
| Premix^1^ | 1.00 |
| Total | 100.0 |
| Nutrient levels |  |
| Metabolizable energy (calculated), kcal/kg | 2,600 |
| Crude protein (calculated/analyzed) | 16.5/16.4 |
| Total phosphorus (calculated/analyzed) | 0.51/0.48 |
| Calcium (calculated/analyzed) | 3.50/3.65 |

^1^ The premix provided the following per kilogram of diets: vitamin A 8000 IU, vitamin D_3_ 1600 IU, vitamin E 30 mg, vitamin K_3_ 1.5 mg, vitamin B_1_ 4 mg, vitamin B_2_ 13 mg, vitamin B_6_ 6 mg, vitamin B_12_ 0.02 mg, biotin 0.15 mg, pantothenic acid 15 mg, nicotinic acid 20 mg, folic acid 1.5 mg, Cu (as copper sulfate) 8 mg, Fe (as ferrous sulfate) 80 mg, Mn (as manganese sulfate) 60 mg, Zn (as zinc sulfate) 80 mg, I (as potassium iodide) 0.35 mg, Se (as sodium selenite) 0.3 mg, choline chloride 800 mg, phytase 200 mg

**Table S2** Primers used for RT-qPCR

| **Genes** | **Prime sequence (5′→3′)** | **NCBI number** |
| --- | --- | --- |
| *NRF2* | F: AGAAAACGCTGAACCACCAATC | NM_205117.2 |
|  | R: GCTGGGTGGCTGAGTTTGATTA |  |
| *HIF1α* | F: GAAGTCAAGAGATGCAGCCAGGTG | NM_204297.2 |
|  | R: GGTCAGCCTCATAATGGATGCCTTG |  |
| *NOX1* | F: TGGACGGAGCACATCATTG | NM_001101830.3 |
|  | R: AGGCAAGCAGGTCATTGAAC |  |
| *HSPB1* | F: GAGATCACCGGCAAACACG | NM_205290.2 |
|  | R: TGATCTCGGATGACTGGATG |  |
| *MGST2* | F: TGACTTAGCTCTGCTCGCTG | XM_001234229.7 |
|  | R: CCAACGTTGAAGCCCAGGTA |  |
| *SOD2* | F: TTGTGATCCATGAGCAGGAA | XM_040669203.2 |
|  | R: TTGTTGCAGATCCCAATCAC |  |
| *GPX3* | F: AGGAGTACATCCCCTTCCGA | NM_001163232.3 |
|  | R: TAGGGCCCCAGCTCATTTG |  |
| *GSR* | F: TCCTGACTACGGCTTCGAGA | XM_015276627.4 |
|  | R: AACTTGCCGTAACCACGGAT |  |
| *VEGFA* | F: GAAGCCCAACGAAGTTATCAAA | NM_205042.3 |
|  | R: ACATCCACAGGGACACATTCTA |  |
| *FLT1* | F: TCCACATCGGCCATCATCTG | NM_204252.2 |
|  | R: CCACGGGCTCAATACCCTTT |  |
| *KDR* | F: CTGAAGGTGCACTCCTCCTC | NM_001004368.2 |
|  | R: CAAGTATGGCTCAACGCAGA |  |
| *ANGPT1* | F: ACAAAAGCGGCGTCTACACT | NM_001199447.4 |
|  | R: AGCCAGTGTTCACCTGATGG |  |
| *ECM1* | F: ATGACATCCCATACCCACGG | XM_040690784.2 |
|  | R: ACTCCAGACTGCTGTTGTGG |  |
| *ITGa5* | F: CTCCAACTACCCCGAGTACT | XM_046904986.1 |
|  | R: CACCGAATAGCCCATATAAC |  |
| *β-ACTIN* | F: AATCAAGATCATTGCCCCACCT | NM_205518.1 |
|  | R: TGGGTGTTGGTAACAGTCCG |  |

*NRF2*, nuclear factor E2-related factor 2; *HIF1*α, hypoxia inducible factor 1 subunit alpha; *NOX1*, NADPH oxidase 1; *HSPB1*, heat shock protein family B member 1; *MGST2*, microsomal glutathione S-transferase 2; *SOD3*, superoxide dismutase 3; *GPX3*, glutathione peroxidase 3; *GSR*, glutathione-disulfide reductase; *VEGFA*, vascular endothelial growth factor A; *FLT1*, vascular endothelial growth factor receptor 1; *KDR*, vascular endothelial growth factor receptor 2; *ANGPT1*, angiopoietin 1; *ECM1*, extracellular matrix protein 1; *ITGa5*, integrin alpha 5

**Table S3** Comparison of production performance between high- and low-producing laying hens at 50 weeks of age

| **Items^1^** | **LP50** | **HP50** | ***P*-value** |
| --- | --- | --- | --- |
| Laying rate, % | 76.7 ± 2.0 | 100.0 ± 0.0 | < 0.001 |
| FCR, g feed/g egg | 2.51 ± 0.07 | 2.07 ± 0.03 | < 0.001 |
| ADFI, g/d/ | 114.8 ± 1.3 | 117.9 ± 1.2 | 0.090 |
| Average egg weight, g | 60.1 ± 1.0 | 57.0 ± 0.8 | 0.028 |

^1^ HP50 and LP50: high- and low-production hens at 50 weeks of age. ADFI: Average daily feed intake; FCR: Feed conversion ratio. Data are presented as mean ± SEM (*n* = 10). Statistical comparisons were performed using an independent samples t-test, with statistical significance set at *P* < 0.05

**Table S4** Comparison of production performance between high- and low-producing laying hens at 75 weeks of age

| **Items^1^** | **LP75** | **HP75** | ***P*-value** |
| --- | --- | --- | --- |
| Laying rate, % | 64.4 ± 2.7 | 96.0 ± 0.7 | < 0.001 |
| FCR, g feed/g egg | 2.86 ± 0.20 | 2.00 ± 0.04 | < 0.001 |
| ADFI, g/d | 109.8 ± 0.9 | 114.2 ± 1.5 | 0.022 |
| Average egg weight, g | 62.9 ± 1.4 | 60.0 ± 1.0 | 0.061 |

^1^ HP75 and LP75: high- and low-production hens at 75 weeks of age. ADFI: Average daily feed intake; FCR: Feed conversion ratio. Data are presented as mean ± SEM (*n* = 10). Statistical comparisons were performed using an independent samples *t*-test, with statistical significance set at *P* < 0.05


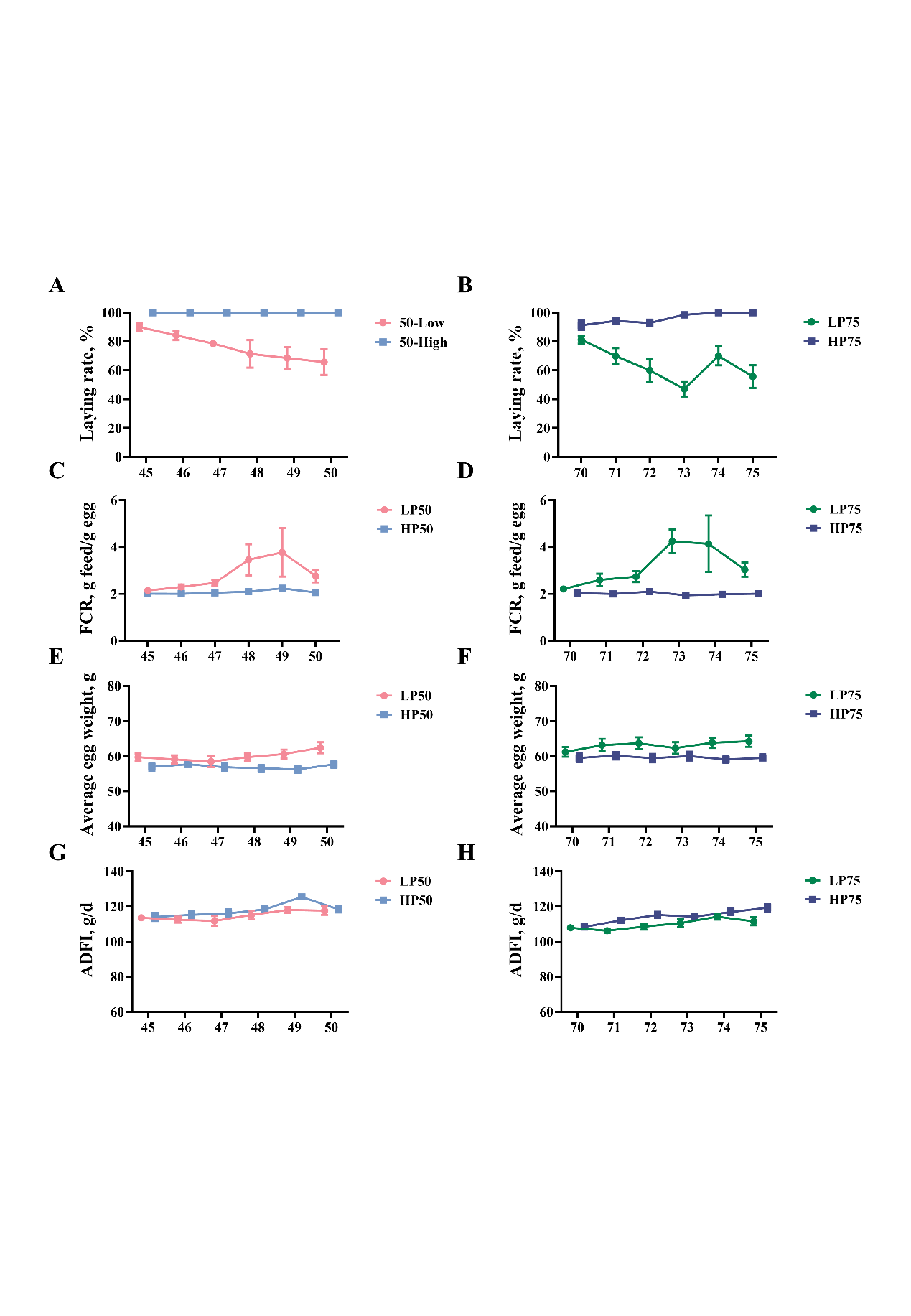


**Fig. S1** Dynamic production performance of high- and low-production laying hens during the 6-week screening period. Individual production data were recorded from 45 to 50 weeks of age (left panels: A, C, E, G) and from 70 to 75 weeks of age (right panels: B, D, F, H). **A** and **B** Laying rate (%). **C** and **D** Feed conversion ratio (FCR, g feed/g egg). **E** and **F** average egg weight (g). **G** and **H** Average daily feed intake (ADFI, g/d). Hens were stratified into high-production (High) and low-production (Low) groups using an unsupervised K-means clustering approach based on their 42-day cumulative performance. Data are presented as means ± SEM (*n* = 10 per group). The consistent divergence between clusters across the 6-week period validates the stability of the selected reproductive phenotypes


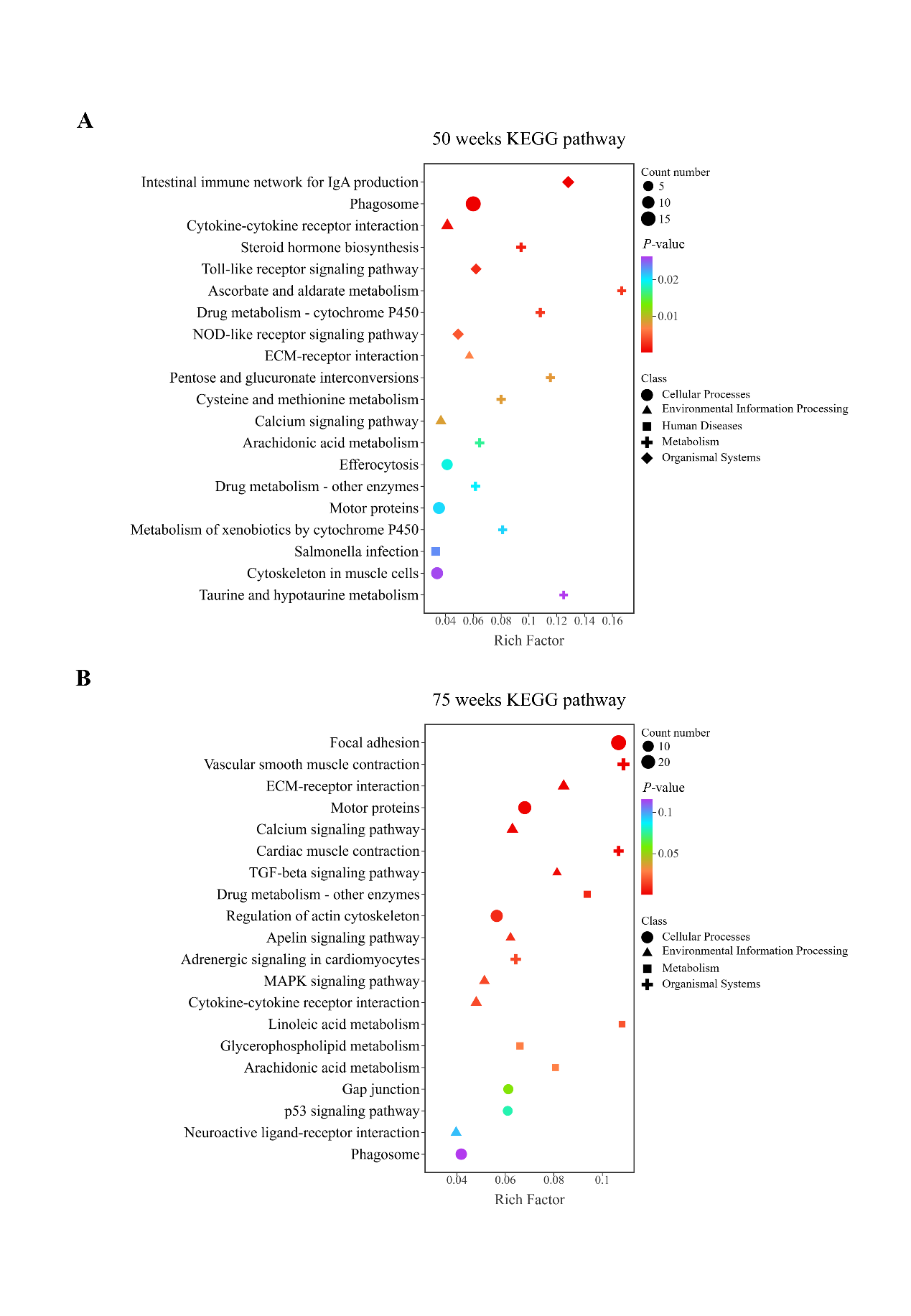


**Fig. S2** KEGG pathway enrichment analysis of DEGs. **A** The group of HP50 vs. LP50, **B** The group of HP75 vs. LP75. DEGs, differentially expressed genes; HP, high production; LP, low production


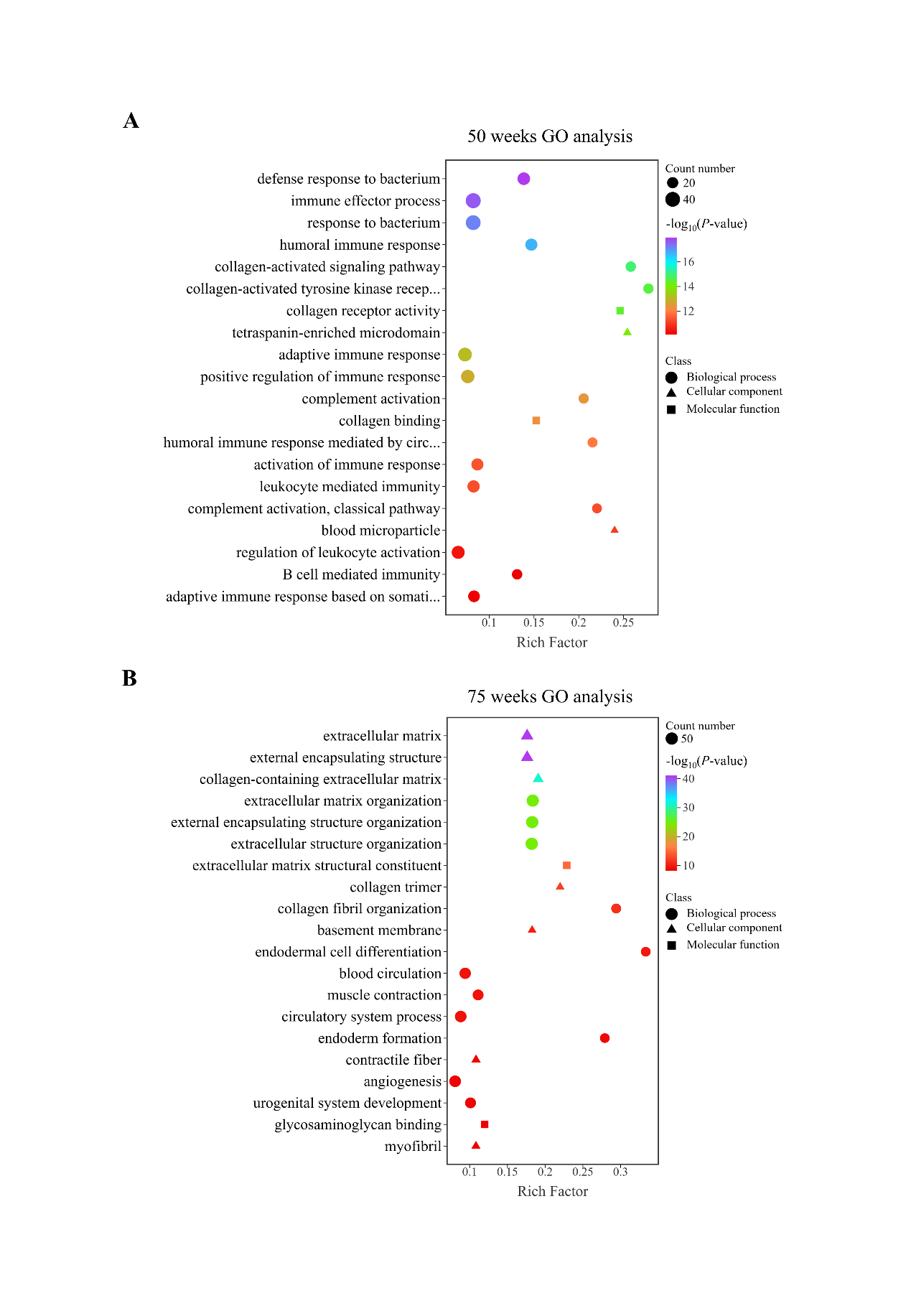


**Fig. S3** GO enrichment analysis of DEGs. **A** The group of HP50 vs. LP50. **B** The group of HP75 vs. LP75. DEGs, differentially expressed genes; HP, high production; LP, low production
